# Mining of Wheat *Pm2* Alleles for Goal-Oriented Marker-Assisted Breeding

**DOI:** 10.3389/fpls.2022.912589

**Published:** 2022-05-12

**Authors:** Ziyang Yu, Luning Xiao, Fuyu Su, Wei Liu, Fuyi Luo, Ran Han, Yanjun Mu, Wenjing Zhang, Liru Wu, Xiao Liang, Nina Sun, Linzhi Li, Pengtao Ma

**Affiliations:** ^1^College of Life Sciences, Yantai University, Yantai, China; ^2^Institute of Grain and Oil Crops, Yantai Academy of Agricultural Sciences, Yantai, China; ^3^Dezhou Agricultural Technology Extension and Seed Industry Center, Dezhou, China; ^4^Crop Research Institute, Shandong Academy of Agricultural Sciences, Jinan, China

**Keywords:** wheat, powdery mildew, *Pm2*, MAS, diagnostic KASP marker

## Abstract

Powdery mildew of wheat, caused by *Blumeria graminis* f. sp. *tritici* (*Bgt*), is a devastating disease that seriously reduces yield and quality worldwide. Utilization of plant resistance genes is an attractive and effective strategy for controlling this disease. Among the reported powdery mildew (*Pm*) resistance genes, *Pm2* exhibits a diverse resistance spectrum among its multiple alleles. It has been widely used in China for resistance breeding for powdery mildew. To mine more *Pm2* alleles and clarify their distribution, we screened 33 wheat cultivars/breeding lines carrying *Pm2* alleles from 641 wheat genotypes using diagnostic and *Pm2*-linked markers. To further investigate the relationships within the *Pm2* alleles, we compared their resistance spectra, polymorphism of marker alleles and gene sequences, and found that they have identical marker alleles and gene sequences but diverse resistance spectra. In addition, the diagnostic kompetitive allele-specific PCR (KASP) marker, *YTU-KASP-Pm2*, was developed and was shown to detect all the *Pm2* alleles in the different genetic backgrounds. These findings provide valuable information for the distribution and rational use of *Pm2* alleles, push forward their marker-assisted breeding (MAS), and hence improve the control of wheat powdery mildew.

## Introduction

Common wheat (*Triticum aestivum* L.) is an important grain crop that provides 20% of the world’s food energy and 20% of its protein. Consequently, it is a major contributor to global food security (Hickey et al., 2019). However, wheat yield and quality are seriously affected by a variety of diseases, including powdery mildew caused by *Blumeria graminis* f. sp. *tritici* (*Bgt*; Costanzo and Bàrberi, 2014 Li et al., 2019). This disease can typically decrease wheat yield by 10%–15%, and up to 50% in serious cases (Morgounov et al., 2012; Xu et al., 2015). Several measures have been taken to control this disease, including the spraying of chemical agents and the use of resistance genes, with the latter being considered to be the most effective and the most environmentally acceptable (Zhang et al., 2019). However, to achieve control using resistance genes, both abundant powdery mildew resistance (*Pm*) genes/alleles and diversified donors are the perquisites for developing elite cultivars with high and/or durable resistance to powdery mildew (Laroche et al., 2019).

To date, more than 100 *Pm* genes/alleles (*Pm1*–*Pm68*, *Pm8* is allelic to *Pm17*, *Pm18* = *Pm1c*, *Pm22* = *Pm1e*, *Pm23* = *Pm4c*, and *Pm31* = *Pm21*) have been identified at 63 loci from common wheat and its relatives, showing that there are abundant genetic resources for controlling wheat powdery mildew (McIntosh et al., 2020; He et al., 2021). However, most of these documented *Pm* genes/alleles cannot be directly used in wheat breeding due to observable linkage drag, adverse pleiotropism, and competition lag. For example, when the gene *Pm16*, derived from *Triticum dicoccoides* and which exhibits broad spectrum resistance to different *Bgt* isolates, was introgressed into wheat backgrounds, it caused up to 15% yield loss during production (Summers and Brown, 2013). Similar yield reductions have been shown for other *Pm* genes derived from wheat relatives and landraces (Xu et al., 2015).

Even for the genes with no linkage drag, evolving *Bgt* variants are another challenge (An et al., 2019). For example, *Pm8* has been a widely used gene in breeding for resistance to powdery mildew. It is also an example of an extremely successful introgression of an elite alien gene from rye into common wheat (Kaur et al., 2017). Unfortunately, *Pm8* has successively lost its resistance due to the co-evolution of pathogen virulence with host resistance (Chai et al., 2005). Clearly, the breeding value of the *Pm* genes depends not only on their effectiveness at disease control, but also on the agronomic performance of their donor (Ma et al., 2015a,b, 2018). Identification and utilization of *Pm* genes with no linkage drag offer an attractive prospect for the rapid improvement of resistance to powdery mildew in wheat.

Conventional breeding has made enormous contributions to resistance breeding in the past (Li et al., 2019, 2022), but marker-assisted selection (MAS) is currently considered to be the most effective way to accurately transfer targeted genes/loci (Li et al., 2019). To improve powdery mildew resistance, MAS has been used with more than 30 *Pm* genes in wheat breeding (Shah et al., 2018). In MAS, the key point is the development and screening of molecular markers that can efficiently trace the targeted genes. Various kinds of markers have been used in MAS, such as expressed sequence tags (EST), sequence-tagged sites (STS), and simple sequence repeat (SSR) markers based on gel electrophoresis detection (Li et al., 2019). With the development of high throughput detection platforms, kompetitive allele-specific PCR (KASP) markers have begun to be used in MAS (Makhoul et al., 2020). KASP was developed based on SNPs in alleles, and enables high-throughput, gel-free screening of markers.

The *Pm2* gene, derived from *Aegilops tauschii*, has been used in resistance breeding for powdery mildew worldwide (Pugsley and Carter, 1953; Li et al., 2011). In our lab, we have identified a series of *Pm2* alleles from different genotypes, including various wheat cultivars/breeding lines, indicating that *Pm2* is a promising *Pm* gene (Gao et al., 2022). Although several *Pm2* alleles no longer exhibit resistance to some *Bgt* isolates, there are still other *Pm2* alleles that confer high resistance to powdery mildew in specific genotypes (Ma et al., 2015a,b, 2018). To maximize the effectiveness of *Pm2* in resistance breeding, in this study we aimed to: (1) mine more *Pm2* alleles and survey their distribution in wheat cultivars/breeding lines; (2) evaluate the resistance of different *Pm2* alleles for their rational utilization in different genetic backgrounds and wheat production regions; and (3) develop diagnostic KASP markers to accelerate the transfer of *Pm2* alleles into breeding lines.

## Materials and Methods

### Plant Materials

Six hundred and thirty-nine Chinese wheat cultivars/breeding lines and two wheat cultivars from New Zealand (Supplementary Table S1) were inoculated with *Bgt* isolate E09 for screening resistant genotypes. Twenty-two wheat cultivars/breeding lines (Shimai 22, Hanmai 13, Shixin 633, Taimai 1918, Tainong 18, Shannong 15,381, Taitianmai 118, Zhongxin 7503, Jimai 52, Jimai 61, Yannong 21, Youxuan 134, GY13029, Xinshiji 156, Shi 6609, Jinhe 13–205, Huixianhong, Mingxian 169, 12CA49, GY16022, GY16011, and 12CA49), which are susceptible to all the *Bgt* isolates tested were used as susceptible parents to cross with the resistant genotypes screened from the 641 wheat genotypes (Supplementary Table S1), to conduct F_2_ and F_2:3_ segregating populations for genetic analysis and molecular detection of their *Pm* genes. Susceptible cultivar Huixianhong was used as the susceptible control for phenotypic assessment. Ulka/8*Cc, which carries the known *Pm2a* gene (Sun et al., 2015a,b), was used as the resistant control.

### Reactions to Different *Bgt* Isolates

At the seedling stage, the *Bgt* isolate E09, a dominant *Bgt* isolate in North China, was used to inoculate the 641 wheat cultivars/breeding line (Supplementary Table S1). Additionally, the *Pm2* donors along with Ulka/8*Cc and Huixianhong were tested for their seedling reaction patterns to eight other *Bgt* isolates (A3, A10, A45, E15-1, E18, E20, E21, and E32) with different avirulence/virulence patterns and from different wheat production regions of China. The susceptible seedlings inoculated with an individual isolate were put in independent glass tubes to avoid cross infection. Five seeds of each genotype were sown in a 72-cell rectangular tray and put in an independent growth chamber to be infected with a *Bgt* isolate. When the seedlings had grown to the one-leaf stage, they were inoculated with fresh conidiospores previously cultivated on Huixianhong seedlings. At this time, the growth chambers were set at 100% humidity at 18°C for 24 h, after which the growing condition was set at 14 h light at 22°C and 10 h of darkness at 18°C. Inoculations were repeated twice in the following 2 days to ensure full infection. Infection types (ITs) were recorded when the spores were fully developed on the first leaves of Huixianhong seedlings based on the standard described by An et al. (2019), where ITs 0, 0; 1, 2, 3, and 4 are regarded as immune, hypersensitive, highly resistant, moderately resistant, moderately susceptible, and highly susceptible, respectively.

To determine the inheritance of powdery mildew resistance in the resistant genotypes, *Bgt* isolate E09 was selected to inoculate the resistant and susceptible parents and their F_1_, F_2_, and F_2:3_ progenies for genetic analysis. After phenotypic evaluation, the numbers of resistance and susceptible plants were counted, and then a goodness-of-fit assessment was performed to determine the resistant/susceptible ratio using a Chi-squared (χ^2^) test. The deviations of the observed phenotypic data from the theoretically expected segregation ratios were then evaluated using the SPSS 16.0 software (SPSS Inc., Chicago, United States) at *p* < 0.05.

### Marker Analysis

Total genomic DNAs (gDNAs) of all the F_2:3_ families along with their parents were isolated after phenotypic evaluation using the TE-boiling method (He et al., 2017). For each population, equal amounts of gDNAs from 10 random homozygous resistant and 10 random homozygous susceptible F_2:3_ families were pooled to construct resistant and susceptible DNA bulks, respectively. The *Pm2*-linked marker *Cfd81* (Ma et al., 2016) and the *Pm2*-diagnostic marker *Pm2b-map-3* (Jin et al., 2021) were tested for polymorphisms between the resistant and susceptible parents and bulks. The polymorphic markers were genotyped on the corresponding F_2:3_ families. PCR amplifications and visualizations were as described by Ma et al. (2016).

### Homology-Based Cloning of the *Pm2* Alleles

Total RNA from each of the genotypes with *Pm2* alleles was extracted using the Spectrum Plant Total RNA kit (Sigma-Aldrich, Shanghai, China) following the manufacturer’s recommendations. Then, they were quantified by measuring absorbance at the wavelengths of 260 and 280 nm using a Nano Drop 1000 spectrophotometer (Thermo Scientific, Shanghai, China). High quality RNA was treated by Promega DNase I and then used for cDNA synthesis using Invitrogen SuperScript-II reverse transcriptase following the manufacturer’s guidelines. Based on the report of the cloning of *Pm2a* (Sánchez-Martín et al., 2016), the primer pairs JS320 (Forward: 5′-3′: ACGATGATGTGAATCTTCCGTG) and JS305 (Reverse 5′-3′: AATGATAGCATGCATTTGGAG) were used to amplify the first exon of the *Pm2* alleles identified in this study. Then, a nested PCR using the primer pairs JS314 (Forward: 5′-3′: TTTTCGCGGTATTGCTGGTG) and JS315 (Reverse 5′-3′: ACCTCCTGTCATCGGTTCAC) was performed to obtain the final sequence of the first exon. For amplifying the second and third predicted exons of the *Pm2* alleles, primer pairs JS350 (Forward: 5′-3′: CCCTCCTCCTTGAAGAATCTGA) and JS313 (Reserve: 5′-3′: GCACAAACTCTACCCTGTTCC) were used. Finally, they were sequenced using Sanger sequencing and compared with that of the reported *Pm2a* (GenBank: LN999386.1; Sánchez-Martín et al., 2016).

### Development of a Diagnostic KASP Marker for MAS

The sequences of the cloned *Pm2* alleles were used to compare with the reference genome of Chinese Spring (v2.1, http://202.194.139.32/). Distinctive SNPs were identified after comparing the *Pm2* sequences to the A, B, and D genomes of Chinese Spring, which does not carry *Pm2* and is susceptible to powdery mildew. Sequences of 100 bp upstream and downstream of the distinctive SNPs were acquired and used for KASP development using both the Polymarker website^1^ and Premier 5 software.^2^ The amplification sequences of the primers were aligned once again with the reference genome of Chinese Spring in the *Triticeae* Multi-omics Center (http://202.194.139.32/) to ensure specificity of the sequences. The primers were then used to genotype all the F_2:3_ families carrying *Pm2* alleles to confirm their polymorphisms. The diagnostic KASP marker was then used to genotype the breeding populations of *Pm2* donors and susceptible cultivars. Combined with the phenotype against *Bgt* isolate E09, the diagnostic KASP marker was confirmed once again.

Genotyping using KASP primers was performed on a Bio-Rad CFX real-time PCR system (Bio-Rad Laboratories, Inc., CA, United States) with a final volume of 20 μl containing 6.00 μl of gDNA (~250 ng), 11.20 μl of 2 × KASP Master Mix (provided by LGC), 0.34 μl of primer mix (balanced mix of three pairs of primers for each marker), and 2.46 μl ddH_2_O. The amplification procedure was set as follows: 94°C for 15 min, followed by 10 touchdown cycles of 94°C for 20 s, 64°C to 58°C (decreasing 0.6°C per cycle), and 38 cycles of regular amplification (94°C for 20 s and 58°C for 60 s), and the final fluorescence was detected at 20°C using Bio-Rad CFX Manager 3.1 software (Bio-Rad Laboratories, Hercules, CA, United States).

## Results

### Screening of Resistant Genotypes and Inheritance Analysis

When inoculated with the *Bgt* isolate E09 at the seedling stage, 43 of the 641 accessions were resistant with ITs 0–2 (Table 1). The 43 resistant accessions were then crossed with susceptible cultivars to produce F_1_ hybrids, F_2_ populations, and F_2:3_ families (Table 1). The F_1_ plants of every hybridized combination all showed resistant phenotypes with ITs 0–2, suggesting dominant inheritance of the powdery mildew resistance in these accessions. Of their F_2_ populations, 40 fitted the expected ratios of 3:1 (resistant: susceptible individuals) for monogenic segregation using the same *Bgt* isolate, suggesting that a single dominant gene may be involved in the powdery mildew resistance of these accessions (Table 1). The 43 F_2_ populations were then transplanted in the field to produce F_2:3_ families to further confirm these results and validate the genotypes of the resistant F_2_ plants. The results showed that 37 F_2:3_ families fitted the expected ratios of 1:2:1 (Homozygous resistant: segregating: homozygous susceptible families; Table 1), confirming that a single dominant gene is involved in the powdery mildew resistance of these accessions. For three populations (Xinong 198 × Yannong 21, GQ17020 × Yannong 21, and GQ17014 × Yannong 21), although their F_2_ segregation ratio fitted the monogenic segregation of 3:1, their F_2:3_ segregation ratio did not fit the monogenic segregation of 1:2:1, suggesting that the powdery mildew resistance in these three accessions may not be controlled by a single dominant gene.

**Table 1:** Segregation ratios of F_2_ and F_2:3_ generations of resistant genotypes and different susceptible cultivars following inoculation with *Blumeria graminis* f. sp. *tritici* (*Bgt*) isolate E09 at the seedling stage.

Resistance parents	Susceptible parents	Segregation ratio/F_2_		χ^2^	*p* Value	Segregation ratio/F_2:3_			χ^2^	*p* Value
		Resistant	Susceptible			Homozygous resistant	Segregating	Homozygous susceptible		
Shannong 05-66	Shimai 22	114	40	0.03	0.85	41	77	42	0.24	0.89
Lande 677	Hanmai 13	121	53	2.84	0.12	45	80	55	3.33	0.19
Zhongxinmai 77	Shimai 22	133	33	2.05	0.15	52	85	35	3.38	0.18
Shimai 24	Taimai 1918	145	44	0.21	0.64	60	89	46	3.49	0.17
FC009	Tainong 18	139	46	0.002	0.97	57	86	48	2.74	0.25
HengH13guan26	Shannong 15,381	66	25	0.30	0.59	23	43	25	0.36	0.83
GQ16002	Taitianmai 118	69	26	0.28	0.59	23	46	26	0.28	0.88
HengHBguan 26	Zhongxin 7,503	77	22	0.41	0.52	28	49	22	0.74	0.69
LS4695	Jimai 61	91	28	0.14	0.71	30	61	28	0.14	0.93
LPM8	Huixianhong	77	26	0.003	0.95	26	51	26	0.01	0.99
Shengmai 127	Huixianhong	94	24	1.37	0.24	31	63	24	1.37	0.50
Jimai 416	Huixianhong	74	22	0.22	0.64	27	47	22	0.56	0.75
Zhongmai 570	Huixianhong	81	26	0.28	0.87	34	47	26	2.78	0.25
Heng 14-K2-3	Huixianhong	116	34	0.44	0.51	36	80	34	0.72	0.70
GQ17023	Huixianhong	97	35	0.16	0.69	31	66	35	0.24	0.89
GQ17018	Huixianhong	95	36	0.43	0.51	35	60	36	0.94	0.63
GQ17006	Yannong 21	85	36	1.45	0.23	30	55	26	0.30	0.86
GQ17015	Yannong 21	94	31	0.01	0.92	33	61	31	0.14	0.93
GQ17053	Yannong 21	41	12	0.16	0.69	12	29	12	0.47	0.79
CH7102	Shixin 633	112	28	1.87	0.17	38	74	28	1.89	0.39
SH3556	Huixianhong	83	25	0.20	0.66	28	55	25	0.20	0.90
HB133-4	Youxuan 134	49	18	0.12	0.72	15	34	18	0.28	0.88
NZ8	Mingxian 169	95	30	0.07	0.80	34	61	30	0.33	0.85
NZ13	Mingxian 169	60	15	1.00	0.32	19	41	15	1.08	0.58
GQ16042	GY13029	84	26	0.11	0.74	29	55	26	0.16	0.92
GQ16010	Xinshiji 156	117	38	0.19	0.89	40	77	38	0.06	0.97
GQ16018	12CA49	69	20	0.30	0.58	24	45	20	0.37	0.83
GQ16031	Huixianhong	93	29	0.10	0.75	33	60	29	0.30	0.86
FC0015	GY16022	94	36	0.50	0.48	31	65	36	0.41	0.82
JieA10Haiping6216	Shi 6609	80	24	0.21	0.65	25	55	24	0.37	0.83
JieA1ShiTa14-7022	Shi 6609	56	15	0.57	0.45	18	38	15	0.61	0.74
Shannong 510659	GY16011	82	25	0.15	0.70	27	55	25	0.16	0.92
JieA13Kemao 60	12CA49	115	33	0.58	0.45	36	67	33	0.16	0.92
Jimai 419	Huixianhong	52	37	13.04	0.0003	10	42	37	25.54	2.84
Xinong198	Yannong 21	70	24	0.01	0.91	9	61	24	13.13	0.001
GQ17025	Huixianhong	72	39	6.08	0.01	10	62	39	16.68	0.0002
GQ17020	Yannong 21	107	47	2.50	0.11	22	85	47	9.78	0.007
GQ17007	Huixianhong	85	52	12.26	0.0005	8	77	52	30.37	2.54
GQ17014	Yannong 21	82	24	0.44	0.50	16	62	24	6.00	0.05
Kenxing 7	Huixianhong	78	23	0.27	0.61	23	55	23	0.80	0.67
Shi CG15-009	Yannong 21	54	15	0.39	0.53	18	36	15	0.39	0.82
Yannong 081531	Jinhe 13–205	84	22	1.01	0.37	28	56	22	1.02	0.60
GY16036	Jimai 52	31	13	0.48	0.49	11	20	13	0.55	0.76

### Screening of Genotypes Carrying *Pm2* Alleles

**Figure 1:**
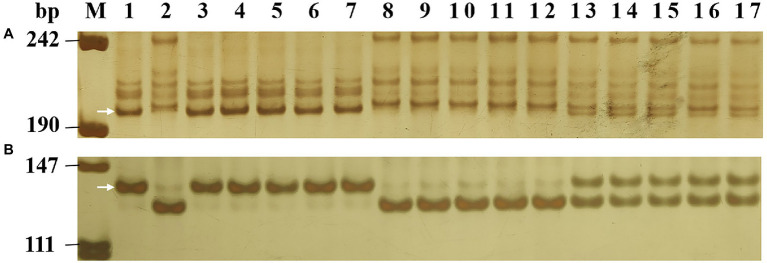
Amplification patterns of the *Pm2*-linked marker *Cfd81* **(A)** and diagnostic marker *Pm2b-map-3* **(B)** in genotyping Lande 677 (*Pm2* donor), Hanmai 13 and random selected F_2:3_ families of Lande 677 × Hanmai 13. Lane M, pUC18 *Msp* I; lane 1, Lande 677; lane 2, Hanmai 13; lanes 3–7, homozygous resistant F_2:3_ families; lanes 8–12, heterozygous F_2:3_ families; and lanes 13–17, homozygous susceptible F_2:3_ families. The white arrows indicate the polymorphic bands in Lande 677.

To identify *Pm2* alleles in the 37 populations that fitted monogenic inheritance, the *Pm2*-linked marker *Cfd81* and the *Pm2*-diagnostic marker *Pm2b-map-3* were used to genotype all 37 segregating populations (Table 2). The results showed that these two markers can amplify consistent polymorphism between resistant parents and bulks in 33 populations. After genotyping the segregating populations, they were proved to be closely linked (*Cfd81*) and co-segregated (*Pm2b-map-3*) with the *Pm* genes in these resistant accessions (Figure 1), suggesting the existence *Pm2* alleles in these resistant accessions. In four other populations (Kenxing 7 × Huixianhong, Shi CG15-009 × Yannong 21, Yannong 081531 × Jinhe 13–205, and GY16036 × Jimai 52), the markers *Cfd81* and *Pm2b-map-3* did not detect *Pm2* alleles, suggesting these populations did not carry *Pm2* alleles.

**Table 2:** Markers used in this study.

Marker	Primer sequence (5′–3′)	References
*Cfd81-F*	AAGATGAACTGCGGCTGAAT	Ma et al., 2016
*Cfd81-R*	CAGATGGACCTCTTCTTCGG	
*Pm2b-map-3-F*	ACCACAACGAACACCAACCT	Jin et al., 2021
*Pm2b-map-3-R*	ACGGGTAACCATCGAGATCA	
*YTU-KASP-Pm2-F*	gaaggtgaccaagttcatgctTGTTGGACGAGAAAAGGAGAAA	Newly developed in this study
*YTU-KASP-Pm2-H*	gaaggtcggagtcaacggattTGTTGGACGAGAAAAGGAGAAC	
*YTU-KASP-Pm2-C*	CAATTCATCTGAGGTGTTGGC	

**Figure 2:**
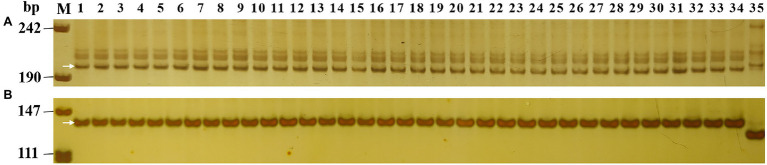
Marker analysis of 33 *Pm2* donors using *Pm2* linked marker *Cfd81* **(A)** and diagnostic marker *Pm2b-map-3* **(B)**. Lane M, pUC18 *Msp* I; lane 1, Huixianhong (control without *Pm2* allele); lane 2, Ulka/8*Cc (control with *Pm2* allele); and lanes 3–35, the *Pm2* donors identified in this study.

To compare the *Pm2* alleles, the 33 *Pm2* donors were initially analyzed using the *Pm2*-linked marker *Cfd81* and the *Pm2*-diagnostic marker *Pm2b-map-3*. The results showed that the 33 *Pm2* donors have the same marker alleles, suggesting consistent genetic diversity in the *Pm2* intervals of these donors (Figure 2). To further dissect the relationship of their *Pm2* alleles, the sequences of the *Pm2* alleles were analyzed after homology-based cloning based on the *Pm2a* sequence. The results indicated that all these *Pm2* alleles were the *Pm2a* haplotype, suggesting that this haplotype has been widely used in resistance breeding for powdery mildew.

### Resistance Spectra of the *Pm2* Donors

**Figure 3:**
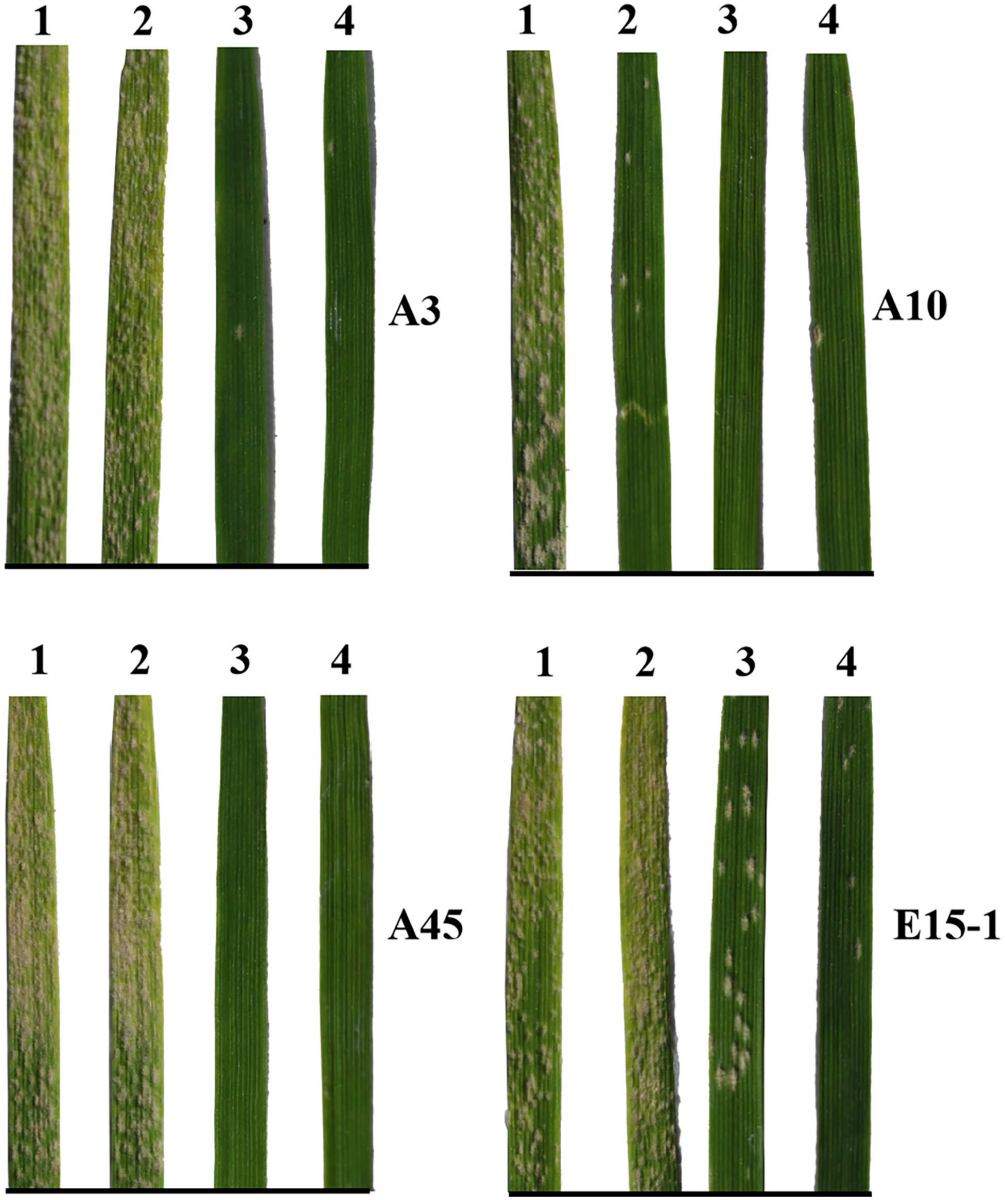
Reaction patterns of several *Pm2* donors to four *Blumeria graminis* f. sp. *tritici* (*Bgt*) isolates, A3, A10, A45, and E15-1, collected from different regions of China using Huixianhong as susceptible control. 1: Huixianhong; 2: Lande 677; 3: Shimai 24; and 4: FC009.

Using nine *Bgt* isolates, including the four highly virulent isolates, E18, E20, E21, and E32, the resistance spectra of the 33 accessions carrying *Pm2* alleles were evaluated. The results showed that the *Pm2* alleles in different genetic backgrounds have different reaction patterns to the nine *Bgt* isolates (Figure 3; Table 3). Some *Pm2* donors showed resistance to all the nine *Bgt* isolates tested, such as Shannong 05–66, GQ16002, GQ17023, and GQ16042. Some *Pm2* donors have poor resistant spectrum, such as GQ16018 and GQ16031 which were susceptible to seven and six *Bgt* isolates. Other *Pm2* donors were susceptible to *Bgt* isolates that were diversified from 1 to 5 ones. This may be related to diversity of genetic backgrounds and/or the interference of other related genes. These data provide a useful reference for breeders in different wheat production regions.

**Table 3:** Reaction patterns of the wheat genotypes with *Pm2* alleles to nine *Blumeria graminis* f. sp*. tritici* (*Bgt*) isolates.

*Bgt* isolates	A3	A10	A45	E15-1	E18	E20	E21	E32	E09
Huixianhong	4	4	4	4	4	4	4	4	4
Shannong 05-66	0	0	0	0	0	0	0	0	0
Lande 677	4	2	4	4	0	3	3	0	1
Zhongxinmai 77	4	2	4	4	0	3	3	0	1
Shimai 24	1	0	0	3	0	3	3	0	1
FC009	1	1	0	2	0	3	3	0	0
HengH13guan26	2	0	1	1	2	1	4	0	0
GQ16002	0	0	0	0	0	0	0	0	0
HengHBguan26	0	0	0	0	2	0	4	0	0
LS4695	2	0	1	1	3	4	4	0	0
LPM8	0	0	0	4	0	4	3	0	0
Shengmai 127	0	3	3	4	0	4	0	0	0
Jimai 416	0	0	0	4	3	4	3	3	0
Zhongmai 570	4	0	4	4	0	4	0	3	0
Heng14-K2-3	0	0	3	4	3	4	0	3	0
GQ17023	0	0	0	0	0	0	0	0	0
GQ17018	0	3	4	4	0	4	0	0	0
GQ17006	4	0	0	4	0	3	0	0	0
GQ17015	0	0	0	0	0	3	3	0	0
GQ17053	0	0	0	4	0	3	3	3	0
CH7102	3	0	-	-	0	0	3	0	0
SH3556	0	0	4	0	0	1	3	3	0
HB133-4	0	3	4	4	0	0	3	4	0
NZ8	0	0	4	4	0	3	3	3	1
NZ13	0	0	3	3	0	3	3	3	1
GQ16042	0	0	0	0	0	0	0	0	1
GQ16010	0	3	0	4	0	4	3	3	1
GQ16018	4	0	3	4	4	4	3	3	1
GQ16031	4	0	3	4	4	4	0	3	1
FC0015	0	0	3	0	4	4	0	3	1
JieA10Haiping6216	0	3	-	-	3	4	3	4	1
JieA1Shita14-7022	3	2	-	-	4	4	0	3	1
Shannong 510659	4	2	-	-	4	4	3	3	1
JieA13Kemao60	0	0	4	4	0	1	3	4	1

Wheat cultivar Huixianhong with no powdery mildew (*Pm*) resistance genes was used as a susceptible control.

Infection types (IT) were scored according to a 0–4 scale, of which 0, 0, 1, and 2 are considered resistant, while those with an IT of 3 or 4 are considered susceptible.

### Evaluation of Markers for MAS

**Figure 4:**
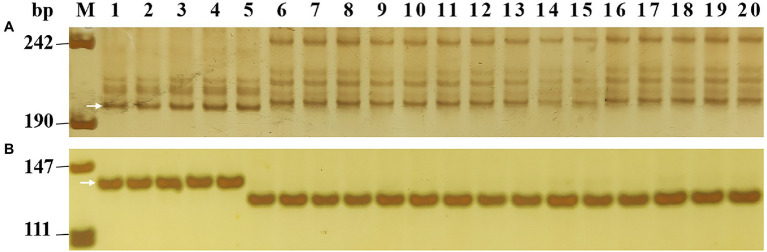
Amplification patterns of *Pm2* linked marker *Cfd81* **(A)** and diagnostic marker *Pm2b-map-3* **(B)** in five *Pm2* donors and 15 susceptible wheat cultivars/breeding lines. M, DNA marker pUC18 *Msp* I; lanes 1–5, five *Pm2* donors with sequential order of Lande 677, LS4695, NZ8, NZ13, and GQ16031; lanes 6–20, wheat cultivars/breeding lines with sequential order of Shimai 22, Taimai 1918, Hanmai 13, Tainong 18, Shannong 15,381, Taitianmai 118, Zhongxin 7503, Jimai 61, Huixianhong, Yannong 21, Shixin 633, Mingxian 169, Xinshiji 156, 12CA49, and Shi 6609. The white arrows indicate the polymorphic bands in *Pm2* donors.

To transfer these *Pm2* alleles to susceptible cultivars using MAS, the gel-based markers *Cfd81* and *Pm2b-map-3* were initially tested for their usefulness in MAS. The results demonstrated that both markers can detect polymorphic genotypes between the *Pm2* donors and the 15 tested susceptible cultivars, suggesting that they can be used for MAS of *Pm2* alleles when these are transferred into these susceptible cultivars by conventional hybridization (Figure 4; Table 4).

**Table 4:** Evaluation of *Pm2*-diagnostic and linked markers on Ulka/8*Cc (*Pm2a* donor) and 15 susceptible wheat cultivars/breeding lines in marker-assisted selection (MAS) breeding.

Wheat genotypes	*Pm2b-map-3*	*Cdf81*	*YTU-KASP-Pm2*
Ulka/8*Cc	+	+	+
Chinese Spring	−	−	−
SH3566	−	−	−
Youxuan134	−	−	−
Mingxian169	−	−	−
GY13029	−	−	−
Xinshiji156	−	−	−
12CA49	−	−	−
Huixianhong	−	−	−
GY16022	−	−	−
Shi 6,609	−	−	−
GY16011	−	−	−
FC0015	−	−	−
Shixin 633	−	−	−
Yannong 21	−	−	−
Tainong 18	−	−	−

“–” indicates that the markers did not amplify the polymorphic products linked to *Pm2* in the relevant cultivar genetic background, and “+” shows that amplification occurred.

**Figure 5:**
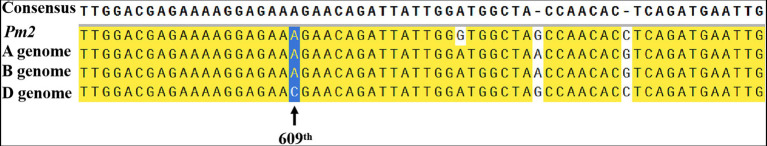
Sequence alignment of the first exon of *Pm2a* and its homologous sequences in the A, B, and D genomes of common wheat Chinese Spring to identify distinctive SNPs for development of diagnostic markers.

**Figure 6:**
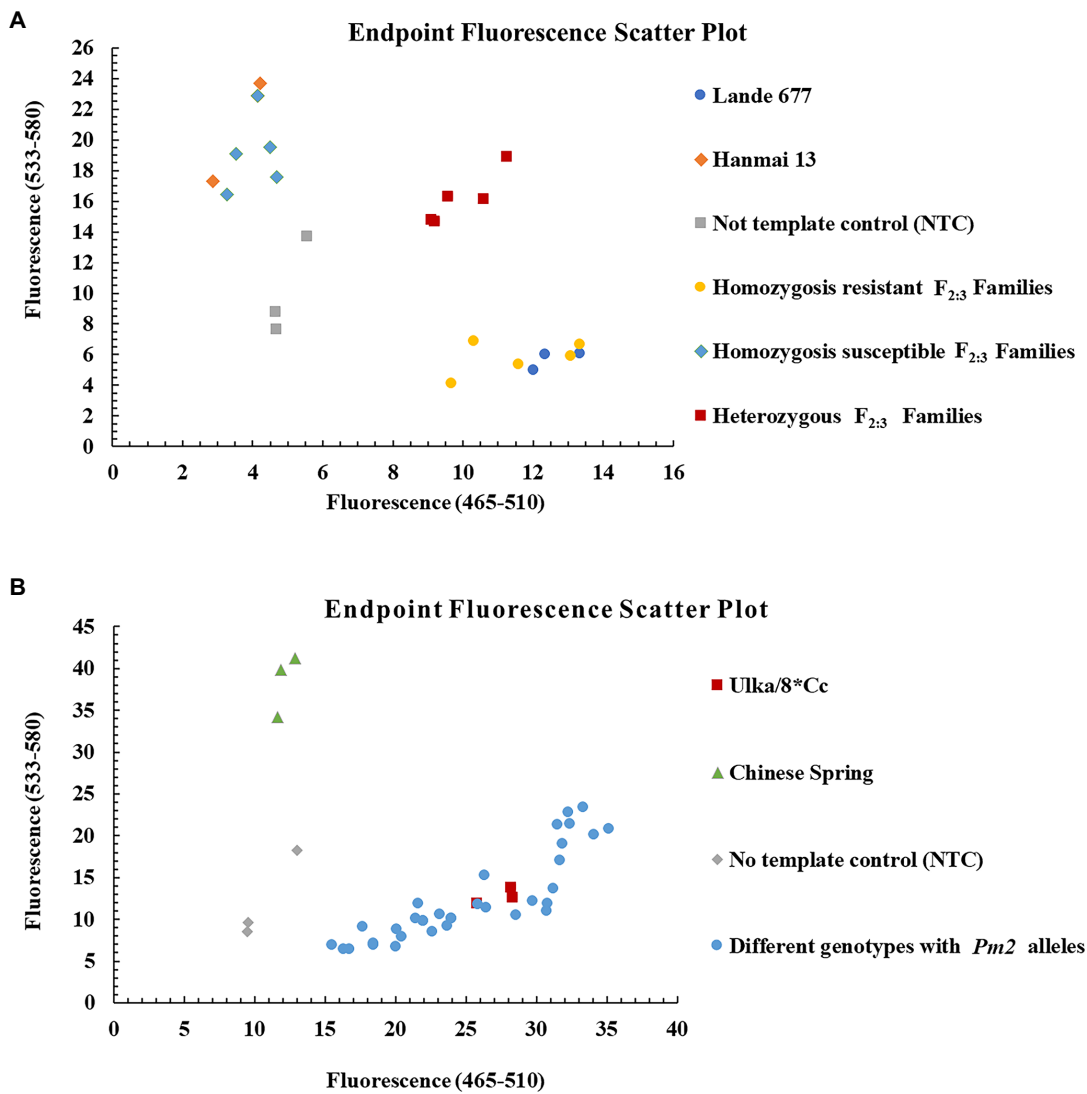
Genotyping results of the *Pm2* diagnostic kompetitive allele-specific PCR (KASP) marker *YTU-KASP-Pm2* for Lande 677 (*Pm2* donor), Hanmai 13 and randomly selected F_2:3_ families of Lande 677 × Hanmai 13 **(A)** and different genotypes with *Pm2* alleles **(B)**.

**Figure 7:**
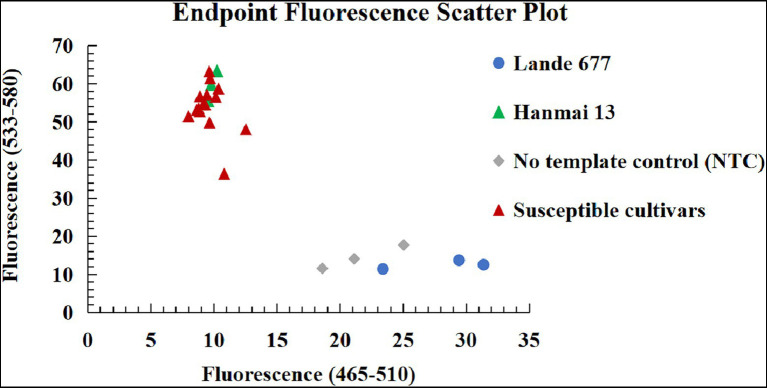
Genotyping results of the *Pm2* KASP marker *YTU-KASP-Pm2* for Lande 677, Hanmai 13 and 15 susceptible wheat cultivars/breeding lines Shimai 22, Taimai 1918, Hanmai 13, Tainong 18, Shannong 15381, Taitianmai 118, Zhongxin 7503, Jimai 61, Huixianhong, Yannong 21, Shixin 633, Mingxian 169, Xinshiji 156, 12CA49, and Shi 6609.

To transfer *Pm2* alleles using a gel-free and high throughput genotyping platform, the diagnostic KASP marker *YTU-KASP-Pm2* was developed to trace the *Pm2* alleles based on the 609th base of the first exon of *Pm2* (Figure 5; Table 2). Using this marker, different *Pm2* donors, plants from the segregating populations and susceptible cultivars without *Pm2* alleles all showed the required genotyping, suggesting *YTU-KASP-Pm2* is an efficient diagnostic marker for *Pm2* (Figure 6; Table 4). *YTU-KASP-Pm2* was then used to genotype the 15 susceptible cultivars to evaluate its suitability for MAS. The result demonstrated that this marker can detect polymorphic genotypes between the *Pm2* donor and each of the tested 15 susceptible cultivars (Figure 7). So, once the *Pm2* allele is transferred into these susceptible genetic backgrounds through conventional hybridization, *YTU-KASP-Pm2* can be used to trace it through the gel-free platform, thus providing a valuable supplement for *Pm2* MAS.

## Discussion

Many *Pm* genes/alleles have been identified that confer resistance to wheat powdery mildew. Among them, only the *Pm* genes that are free of linkage drag and/or adverse pleiotropism have significant potential in resistance breeding. The gene, *Pm2*, was initially identified in the wheat landrace Ulka from the former Soviet Union in 1953 (Pugsley and Carter, 1953). In the 70 years of breeding history using *Pm2*, this gene has had exceptional performance in conferring resistance to powdery mildew. Many wheat cultivars carrying *Pm2* have been bred and used in production, such as Liangxing 66 with *PmLX66* (Huang et al., 2012), Jimai 22 and Jimai 23 with *PmJM23* (Jia et al., 2020), Yingbo 700 with *PmYB* (Ma et al., 2015c), Zhongmai 155 with *PmZ155* (Sun et al., 2015a), Nongda 399 with *MlND399* (Li et al., 2013), Wennong 14 with *PmW14* (Sun et al., 2015b), and Heng 4568 with *PmH4568* (Gao et al., 2022). Apart from the cultivars in production, many breeding lines and landraces have also been shown to carry *Pm2* alleles, including KM2939 with *Pm2b* (Ma et al., 2015a), Niaomai with *Pm2c* (Xu et al., 2015), Wangfengjian 34 with *PmWFJ* (Ma et al., 2015b), CH1357 with *PmCH1357* (Chen et al., 2019), 10V-2 with *Pm10V-2* (Ma et al., 2018), FG-1 with *PmFG* (Ma et al., 2016), Subtil with *PmSub* (Jin et al., 2018), and X3986-2 with *PmX3986-2* (Ma et al., 2014). After 70 years in production, a number of *Pm2* alleles continue to show high and broad-spectrum resistance in some genetic backgrounds, such as 10V-2, YingBo 700, KM2939, and Niaomai (Ma et al., 2015a,c, 2018; Xu et al., 2015). However, other alleles have reduced their ability to confer resistance (Ma et al., 2014, 2016).

It is necessary to identify more *Pm2* donors from wheat cultivars and breeding lines and to also clarify their distribution in different wheat production regions, so that their use can be promoted in different regions. In this study, after wide screening, we identified a large number of *Pm2* donors from 641 wheat cultivars and breeding lines collected from different wheat production regions. This is the first time that the existence of *Pm2* alleles in current wheat breeding lines has been assessed and summarized, data which will contribute to realizing the synergistic improvement of resistance and other agronomic traits. The results revealed that *Pm2* alleles accounted for a very high proportion of the resistance genes in resistant cultivars and breeding lines. This suggests more careful use of this gene in production is necessary, and the best strategy for its use may be in pyramiding it with other resistance genes to develop durable resistance.

Using mutant chromosome sequencing (MutChromSeq), the *Pm2a* allele was cloned (Sánchez-Martín et al., 2016). Furthermore, Chen et al. (2019) cloned *PmCH1357* using map-based cloning and found that *PmCH1357*, *Pm2c*, *PmLX66*, and *MlND399* all have identical sequences to *Pm2a*. However, Manser et al. (2021) identified seven new allelic variations of *Pm2* from 28 *Aegilops tauschii* accessions. This implies that there may have been only one haplotype of *Pm2* that is *Pm2a*, used in resistance breeding since it was first identified in 1953. To confirm this result, we identified additional *Pm2* alleles in the 641 wheat cultivars and breeding lines and found that all of the *Pm2* alleles were identical to haplotype *Pm2a*, further confirming the conservation of the *Pm2a* haplotype in wheat breeding. From this result, we speculate that the new *Pm2* alleles identified in *Aegilops tauschii* could also become valuable contributors to resistance, just as *Pm2a* has been an elite allele used in breeding for more than 70 years and which still confers considerable resistance.

Nevertheless, there are still unsolved issues associated with the *Pm2* locus. We, and others (Ma et al., 2014, 2018; Sun et al., 2015a,b; Jia et al., 2020), have now shown that different *Pm2* donors have significantly different reaction patterns to different *Bgt* isolates, something that is hard to explain based solely on the different genetic backgrounds. We speculate that there may be other unknown associated genes in the different genetic backgrounds which, together, provide the powdery mildew resistance in specific genotypes. We also note that there is still no transgenic evidence to confirm the functionality of the cloned *Pm2* gene, something that is required to clarify this locus.

Irrespective of the unsolved issues associated with the *Pm2* locus, this has not prevented its use in breeding in view of the advantages of this locus in conferring resistance. In wheat resistance breeding, MAS is a rapid and effective method to trace targeted genes in breeding programs, and development of molecular markers for MAS has been the key factor (William et al., 2007; Gupta et al., 2010; Li et al., 2019). Currently, there are two types of breeding markers (gel-based and gel-free), which can meet the needs of different labs in terms of throughput and which are applicable to different platforms (gel-based or gel-free) for marker analysis. Gel-based markers for *Pm2* have been reported in previous studies (Ma et al., 2015a,b,c, 2016, 2018). We verified their suitability in identifying *Pm2* donors in this study, including both linked and diagnostic markers.

With the development of high throughput genotyping platforms, gel-free KASP markers are now also widely used. Compared with the gel-based markers, KASP markers have the advantages of good stability and high throughput, are free of specific fluorescent probes and are low cost (Rasheed et al., 2016). However, no KASP markers suitable for MAS had been reported for the elite *Pm2* gene. To efficiently and accurately transfer and trace *Pm2* alleles using MAS, we developed the diagnostic KASP maker *YTU-KASP-Pm2* based on the presence of a stable SNP between homologous genes in the susceptible cultivars and the *Pm2* donors. This means that the *Pm2* alleles can be detected through two platforms: the gel-based diagnostic marker *Pm2b-map-3* can be used by breeders with only basic screening facilities, and the gel-free marker *YTU-KASP-Pm2* is available to breeders with high-throughput genotyping platforms.

When an elite resistance gene is identified, its breeding value depends not only on its resistance but also on the agronomic performance of the gene donor. Gene donors with poor agronomic performance will greatly limit their utilization in breeding (Summers and Brown, 2013; Liu et al., 2019, 2020). In this study, all the *Pm2* alleles identified were from wheat cultivars or breeding lines. Their donors all have elite yield and agronomic performance and, more importantly, they came from different wheat production regions, which can meet the requirements of different wheat production regions in corresponding resistance breeding. These *Pm2* alleles can be individually used in corresponding ecoregions, and also can be used in pyramiding breeding with other resistance genes for durable resistance. The abundance of *Pm2* donors provides the possibility of screening the optimum pyramiding model in breeding and realizing the dual improvement in both resistance and agronomic traits.

## Conclusion

In conclusion, we have identified 37 *Pm2* alleles from wheat cultivars and breeding lines collected from different wheat production regions and confirmed that they all have the *Pm2a* sequence, but have different reaction patterns to different *Bgt* isolates depending on their specific genetic background. Molecular markers available for MAS were screened and confirmed, and a diagnostic KASP marker *YTU-KASP-Pm2* was identified for use in high-throughput genotyping platforms. Our study has provided valuable information on the distribution and rational use of *Pm2* alleles and will contribute to the control of wheat powdery mildew.

## Data Availability Statement

The original contributions presented in the study are included in the article/Supplementary Material, further inquiries can be directed to the corresponding authors.

## Author Contributions

PM, LL, and NS conceived the research. ZY, LX, and FS performed the experiments. WL, YM, WZ, LW, and XL analyzed the data. FL, RH, NS, and LL performed phenotypic assessment. PM wrote the manuscript. All authors contributed to the article and approved the submitted version.

## Funding

This research was financially supported by the National Natural Science Foundation of China (32072053, 31971874, and 32001544) and Key Technology Research and Development Program of Shandong (2020CXGC010805 and 2021RKY06100).

## Conflict of Interest

The authors declare that the research was conducted in the absence of any commercial or financial relationships that could be construed as a potential conflict of interest.

## Publisher’s Note

All claims expressed in this article are solely those of the authors and do not necessarily represent those of their affiliated organizations, or those of the publisher, the editors and the reviewers. Any product that may be evaluated in this article, or claim that may be made by its manufacturer, is not guaranteed or endorsed by the publisher.

## Footnotes

^1^ http://www.polymarker.info/

^2^ https://primer-premier-5.software.informer.com/
